# An evaluation of government‐recommended stocking systems for sustaining pastoral businesses and ecosystems of the Alpine Meadows of the Qinghai‐Tibetan Plateau

**DOI:** 10.1002/ece3.3960

**Published:** 2018-04-02

**Authors:** Yingxin Wang, Kenneth C. Hodgkinson, Fujiang Hou, Zhaofeng Wang, Shenghua Chang

**Affiliations:** ^1^ State Key Laboratory of Grassland Agro‐Ecosystems Key Laboratory of Grassland Livestock Industry Innovation, Ministry of Agriculture College of Pastoral Agriculture Science and Technology Lanzhou University Lanzhou China; ^2^ CSIRO Land and Water Canberra ACT Australia

**Keywords:** alpine meadow, animal productivity, grasslands, plant species productivity, Qinghai‐Tibetan Plateau, sheep stocking, soil nutrients, stocking policy, stocking regimes

## Abstract

China introduced the “Retire Livestock and Restore Grassland” policy in 2003. It was strengthened in 2011 by additional funding for on‐farm structures. On the Qinghai‐Tibetan Plateau (QTP), fences were erected, livestock excluded from degraded areas, rotational stocking introduced, nighttime shelters were built, forages grown, and seed sown. However, the effectiveness of these actions and their value to Tibetan herders has been questioned. We conducted a sheep stocking experiment for 5 years in an Alpine Meadow region of the QTP to evaluate stocking options recommended by Government. Cold and warm season stocking each at three rates (0, 8, and 16 sheep/ha) and continuous stocking at 0 and 4 sheep/ha were compared. We measured live weights of sheep, plant species richness and evenness, root biomass and carbon (C), nitrogen (N) and phosphorus (P) contents of the 0–10 cm of soil. We found that resting grassland from stocking during the warm season for later cold season stocking significantly reduced plant species richness and evenness and root biomass but not soil C, N, and P. During cold season stocking, live weights of sheep declined whether at a stocking rate of 8 or 16 per ha. In contrast, sheep continuously stocked on grassland at 4 per ha gained weight throughout both the warm and cold seasons and plant species richness and evenness were maintained. Warm season stocking at 8 and 16 sheep/ha increased plant species richness and root biomass but reduced plant species evenness. Resting these alpine grasslands from stocking in the warm season has adverse consequences for plant conservation. Fencing from stocking in the warm season is not justified by this study; all grassland should be judiciously stocked during the warm season to maintain plant species richness. Neither resting nor stocking during the cold season appears to have any adverse consequences but sheltering and in‐door feeding of sheep during the cold season may be more profitable than cold season stocking with use of open nighttime yards.

## 1 INTRODUCTION

Natural grasslands of the world may degrade when over‐stocked (Alkemade, Reid, van den Berg, de Leeuw, & Jeuken, 2013; Steinfeld, Mooney, Schneider, & Neville, 2013). The degradation impacts the economic outcome and sustainability of pastoral businesses and of the people who are living remotely (Andersson, Brogaard, & Olsson, 2011; Bedunah & Angerer, 2012; Noojipady, Prince, & Rishmawi, 2015; Smith et al., 2007). For herders of the Qinghai‐Tibetan Plateau (QTP), the loss of palatable plant species, soil erosion, and poor sheep and yak production limits their ability to improve personal income and to cope with adverse consequences of climate change (Li et al., 2013; Wang, Dong, et al., 2015; Wang, Lassoie, Morreale, & Dong, 2015). Degradation of the Alpine Meadows of the QTP, as assessed by remote sensing, is severe, moderate, or light in 6%, 18%, and 28%, respectively, of the area (Zhao et al., 2015). Here plant species richness has declined, unpalatable, and poisonous plants have increased as has “accelerated” soil erosion (Wang, Dong, et al., 2015; Wang, Lassoie, et al., 2015). Harris (2010), however, argues that “causes of degradation remain uncertain, often because hypotheses have been articulated too vaguely to test.” Causes for degradation are variously attributed; increased rainfall variability from climate change (Chen et al., 2013; Lehnert, Wesche, Trachte, Reudenbach, & Bendix, 2016), high localized herbivory, and soil disturbance from Plateau Pika (*Ochotona curzoniae*) and Plateau Zokor (*Myospalax baileyi*) (Harris, Wang, Smith, & Bedunah, 2015; Pang & Guo, 2017; Smith & Foggin, 1999), road building (Qiu, 2014) and increased domestic stock numbers (Hao, 2008; Qiu, 2007).

The nomadic stocking systems practiced by herders for many centuries are thought to have sustained the functionality of the grasslands (Brondizio & Le Tourneau, 2016; Miller, 1999). In contrast, the policies successively introduced by the Central Government of China since 1950 have been challenged (Harris, 2010; Qiu, 2016; Wang, Dong, et al., 2015; Wang, Lassoie, et al., 2015) on grounds that they are not sustainable. In the nomadic systems that prevailed prior to 1950, stock were periodically moved during the warm seasons. Stock movement was determined by forage availability across the landscapes and a highly regulated social system that required the grassland to be maintained in a productive and functional state (Miller, 1999). From 1950, herders increasingly grazed stock in a collective manner on areas allocated by government using a two‐season stocking system; grazing their stock on mountain slopes in the warm season and in the valleys for the cold season.

In 1980, the Government introduced the “Grassland Household Contract System” policy and the two‐season stocking system was mostly replaced by sedentary systems whereby herders were allocated defined areas of grassland. Unfortunately, the stock of each herder increased in number because personal wealth of herders is determined by the number, and not the condition, of their stock (Cao, Xiong, Sun, Xiong, & Du, 2011). The degradation of the QTP grassland of the QTP, especially of the nonmeadow grasslands, rose from 24.5% in 1980 to 34.5% in 1990 (Li et al., 2013). It was commonly believed that the degradation predisposed an increase in Plateau Zokor and Pika populations (Kang, Han, Zhang, & Sun, 2007) but this has been challenged (Pang & Guo, 2017; Pech, Arthur, Zhang, & Lin, 2007).

In 2003, the Government introduced the “Retire Livestock and Restore Grassland” policy and fences were erected for resting from stocking of grassland in the warm season and for the long‐term exclusion of stock from severely degraded areas, shelters for protecting stock from cold and predators were built, forages for supplementary feeding during the long cold period were grown, and grassland species were sown to renew degraded pasture. Some of these measures received Government financial support. The policy also recommended that rotational stocking, as practiced in other countries (Briske et al., 2008; Welchons et al., 2017), be scientifically evaluated on the QTP and if successful be promoted for adoption by herders (Du, Yan, Chang, Wang, & Hou, 2017; Liu, Li, Ouyang, Tam, & Chen, 2008; Wang, Wang, He, Liu, & Hodgkinson, 2010; Wang, Zhao, Long, & Yang, 2010).

The major problem for stocking businesses on the QTP grasslands is managing yearlong production systems in a grassland with a short growing season (Shang et al., 2014). The relationship between grassland and stock productivity during the warm season is well‐established from grazing experiments conducted in Australia, England, and the USA and synthesized into a global model (Jones & Sandland, 1974). This model is a linear negative relationship between stocking rate and weight gain per animal from which the total stock productivity per unit of grassland is calculated and takes the form of a quadratic relationship. Later, Wilson and MacLeod (1991) reasoned that there would be loss of linearity if degradation occurred and they developed theoretical relationships from the global model of Jones and Sandland to show that the loss of linearity would reduce stock productivity and business profitability. Stocking rate studies on the QTP grasslands, conducted during the warm season, for both yak and sheep, however, have shown no loss of linearity in animal production at higher stocking rates (Dong, Zhao, Wu, & Chang, 2015; Kemp et al., 2013; Miao, Guo, Xue, Wang, & Shen, 2015; Sun, Angerer, & Hou, 2015). However, stocking rates in these experiments were on the conservative side; very high stocking rates in line with common practice were not imposed.

Loss of linearity would be preceded by change in the botanical composition of the grassland or other floristic attributes and/or change in soil properties (Ludwig, Tongway, Freudenberger, Noble, & Hodgkinson, 1997). These are early warning signs of an approaching critical threshold beyond which loss of linearity will occur (Westoby, Walker, & Noy‐Meir, 1989). Comparison of a range of stocked sites in QTP grasslands that differed in perceived degradation status have demonstrated changes in plant species richness and soil attributes (e.g., Wang, Lassoie, et al., 2015; Wang, Dong, et al., 2015; Wen et al., 2013; You et al., 2014). However, in these studies, the stocking histories at the sites were not taken into account. It is possible that differences in perceived degradation arose from a combination of factors such as the co‐occurrence of drought and grazing rather than stocking rate per se. A study by Lu et al. (2017) of many paired grazed and un‐grazed grassland sites on the QTP suggested plant species richness declined in the absence of stocking whereas soil carbon, nitrogen, and microbial biomass increased.

We conducted a sheep stocking experiment to evaluate the appropriateness of the “Retire Livestock and Restore Grassland” policy. The questions evaluated were as follows: (1) Are there any adverse consequences from exclusion of stocking during the warm season to produce “reserved” pasture for cold season stocking, (2) is continuous stocking in the warm and cold seasons an unsustainable practice, (3) is warm season only stocking (with housing of stock in the cold season) a sustainable practice, and (4) is a stocking rate for “optimal” profitability sustainable? Specifically, hypotheses and predictions for each question are (1) plant and soil attributes do not change when there is no stocking during the warm season; removal of large herbivores from natural grassland will not in the midterm change soil attributes because they are slow moving but loss of plant species by rank growth in the warm season lethally shading low growing species is likely, (2) continuous stocking is not sustainable; plant and soil attributes are unaffected by continuous stocking at low stocking rates but quality of sheep would deteriorate during the cold season, (3) warm season only stocking is not a sustainable practice; the cost of feed during the cold season housing of sheep is sufficiently low for the practice to be sustainable, and (4) the optimal stocking rate for profitability is not sustainable; plant and soil attributes are not adversely changed by the stocking rate at which stock weight gain per unit of grassland is at a maximum.

## 2 MATERIALS AND METHODS

### 2.1 Study site

**Figure 1:**
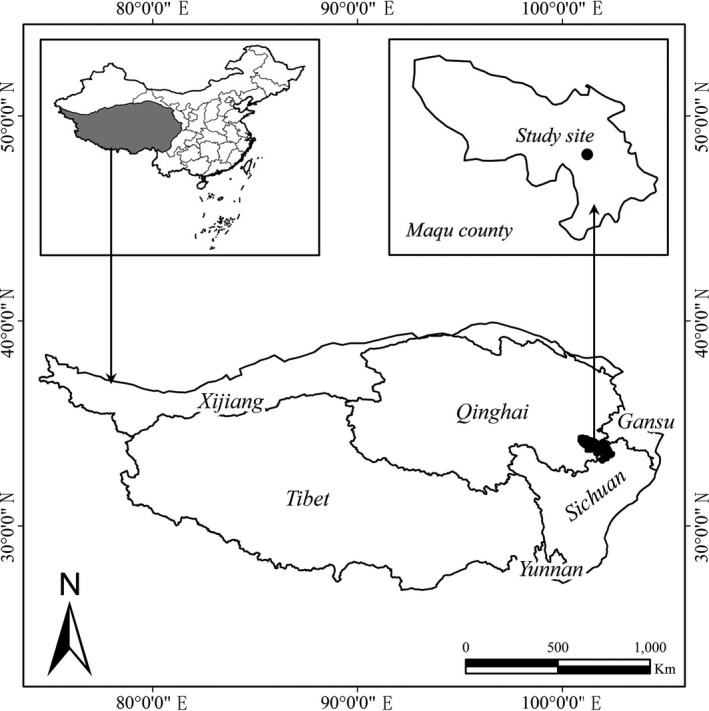
Location of study site on the Qinghai‐Tibetan Plateau. The Plateau is bordered by Sichuan and Yunnan Provinces in the south‐east and by Gansu and Xinjiang Provinces in the north and north‐west

The study site (latitude 33°42′21″N, longitude 102°07′02″E, elevation about 3,600 m a.s.l.) is located on the eastern side of the QTP in the Maqu County, Gannan Prefecture, Gansu Province, China (Figure 1). Here the warm season is from June to September, and the cold season is from October to May. Mean annual temperature is 1.2°C, mean daily temperature is −8.9°C in January and 11.9°C in July. There are on average 270 frost days per year. Mean annual precipitation is 620 mm, falling mostly in July and August. Annual cloud‐free solar radiation is about 2,580 h. Soil type is Alpine Meadow Soil, that is, primarily Mat‐Cryic Cambisols (Chinese Soil Taxonomy Research Group 1995). Vegetation is alpine meadow (Ren et al., 2008) and comprises sedges, grasses, and forbs. Dominant plant species are *Kobresia graminifolia*,* Elymus nutans*,* Agrostis* species, *Poa pratensis, Saussurea* species, and *Anemone* species. The study site had been continuously stocked by yaks for the last 30 years before beginning our sheep stocking treatments. There were Plateau Pika and Plateau Zokor populations at the site during the study but populations were at very low densities.

### 2.2 Sheep stocking treatments

The treatments were:

1. *Warm season stocking* from July to September at two rates of 8 and 16 sheep/ha under rotational stocking;
2. *Cold season stocking* from October to December at two rates of 8 and 16 sheep/ha under rotational stocking;
3. *Warm + cold season stocking* from July to December (warm and cold seasons) at the rate of 4 sheep/ha under continuous stocking and
4. No stocking (*Control*).

Stocking each year began a month after the commencement of the warm season because it was not possible to obtain the young sheep any earlier from local herders and it took several weeks to prepare the animals for grazing. The stocking ended each year in December of the cold season (rather than continuing to May) because work and measurements in this extreme and remote environment were impossible to sustain for the researchers during the last 5 months of the cold seasons.

The terminology used in the paper is the internationally accepted standard terms for grazing lands (Allen et al., 2011).

There were eight sheep in each paddock at any time in the warm, cold, and warm + cold season stocking treatments (Table 1). For the warm and cold seasons, stocking rates of 8 and 16 sheep/ha were in paddock sizes of 1 and 0.5 ha, respectively. For the warm + cold season, the stocking rate of 4 sheep/ha was in a paddock size was 2 ha. The no stocking (Control) treatment was fenced areas of 25 m^2^ in each paddock.

**Table 1:** Key details of each stocking treatment

Grazing treatment	Stocking rate (sheep/ha)	Paddock sizes	Sheep numbers/paddock	Replicates
Warm season grazing	0	25 m^2^	0	6
	8	1 ha	8	6
	16	0.5 ha	8	6
Cold season grazing	0	25 m^2^	0	6
	8	1 ha	8	6
	16	0.5 ha	8	6
Warm + cold season grazing	0	25 m^2^	0	3
	4	2 ha	8	3

**Figure 2:**
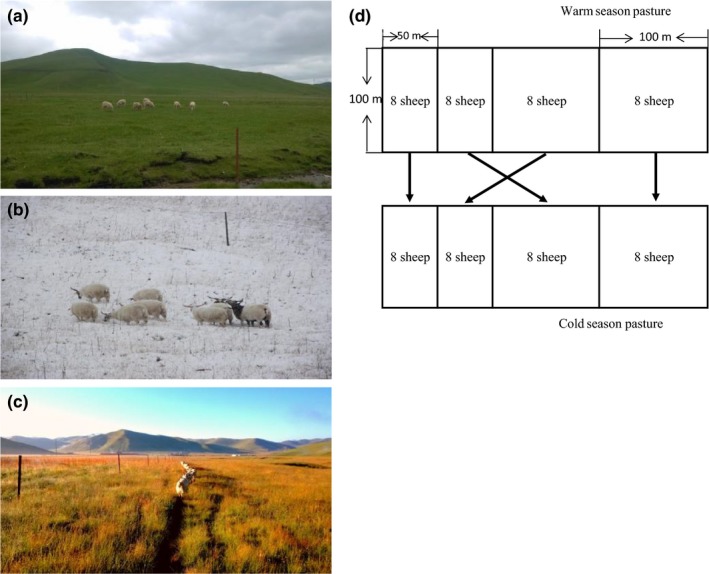
The stocking treatments at the site in Maqu County (Gannan Prefecture, Gansu Province, China) (a) warm season stocking; (b) cold season stocking; (c) warm + cold season stocking; (d) The layout of rotational stocking

Stocking treatment paddocks were fenced in early spring of 2010. There were six replicates of the warm and cold season stocking treatments. Within each replicate, the warm season stocked paddocks were subdivided into three sub‐paddocks and the cold season stocked paddocks were subdivided into two sub‐paddocks as shown in Figure 2. Sheep were moved between the sub‐paddocks every 10 and 15 days in the warm and cold seasons, respectively. There were three replicates of the warm + cold season stocking treatment, and the paddocks were not sub‐divided; the grassland was continuously stocked for 6 months of the year. The experiment was conducted for 5 years and terminated at the end of 2014.

### 2.3 Sheep management

Each year 150 castrated male Tibetan sheep, 5 to 7 months old were purchased in June from nearby herders. Of these, 120 were assigned to the study and the remaining were grazed outside the treatment paddocks and used to replace animals killed by wolves (*Canis lupus*) or disease. In December, the sheep were sold.

Initially, sheep were ear‐tagged, vaccinated, drenched for parasite control with Albendazole (Hanzhong Tianyuan Pharmaceutical Co. Ltd, Shanxi, China) and weighed on two consecutive days. The 120 heaviest sheep were divided into 15 groups of eight sheep with each group having a similar average weight. The members of each group were labeled with specific rump markings. These markings enabled the herder to place the members of each group to assigned paddocks each day. For an “acclimatization” period of 1~2 weeks, the sheep grazed outside the treatment paddocks and had access to mixed‐mineral block and fresh water.

After the “acclimatization” period, the sheep were distributed in their groups to designated paddocks where they had continuous access to mixed‐mineral blocks. Each day in the late afternoon and early morning sheep were herded from the paddocks, given access to stream water and mixed‐mineral blocks, then held overnight in designated compartments of the yard and protected from wolves and thieves. The herder slept in a tent next to the yard.

### 2.4 Data collection

#### 2.4.1 Sheep weights

Each sheep was weighed at the end of each month on two consecutive days. The weight gain per sheep per season was calculated as the difference between the weights at the beginning and the end of the three monthly seasons. Weight gain per hectare was calculated from the number of sheep in each paddock times the average seasonal‐weight gain.

#### 2.4.2 Shoot and root biomass of vegetation

Each month, a 0.25‐m^2^ quadrat was placed in the central region of each sub‐paddock and the shoots of each quadrat were cut and the on‐ground litter removed and bagged together. The soil in the quadrat was sampled in the center with a 10‐cm diameter auger 40 cm long. Soil was removed in 10 cm layers, down to a depth of 40 cm and each layer was separately placed in 0.2 mm mesh bags.

Shoots of each species and the litter were separated, oven‐dried at 65°C for 48 hr and weighed. The total shoot weight was the sum of individual species. Soil samples were air‐dried for 1 month in a glasshouse, and roots were separated from them. The roots were washed free of soil, oven‐dried at 115°C for 48 hr and weighed. In this study, the data from the soil and root samples taken in August of each year are presented.

#### 2.4.3 Shoot biomass of each species

From the shoot biomass of each species in each sample (quadrat), two indices were derived. We used biomass of each species because the plant density was too high for accurate counting of the number of individuals of each species.

*Plant species richness (S)*: This was obtained from a count of the number of plant species in the sample taken from each quadrat area.
*Plant species evenness (E)*: This was calculated from the biomass of each species in a quadrat using the formula proposed by Camargo (1993). *E* is an estimate of the distribution of abundance among species and as such is an important descriptor of a community of plants. A community in which each species is equally abundant has high evenness; a community in which the species differ widely in abundance has low evenness (Smith & Wilson, 1996). *E* is calculated independently of plant species richness and together with *S* is used to compute of plant species diversity. The Camargo evenness index is defined as follows:
  $$E=1-\sum_{i}^{S}\sum_{j=i+1}^{S}\left(\left|Pi-Pj\right|/S\right)$$

where *E*: Camargo evenness index; *Pi*: the proportion of species *i* in the sample; *Pj*: the proportion of species *j* in the sample; *S*: the total number of plant species in the quadrat.

#### 2.4.4 Soil carbon, nitrogen, and phosphorus

The soil samples were air‐dried at room temperature in the laboratory for 30 days and sieved through a 2‐mm sieve. Soil organic carbon (SOC) was measured by Walkey and Black method (Nelson & Sommers, 1982). Total Kjeldahl nitrogen (TN) and total phosphorus (TP) were analyzed using a FIAstar 5000 flow injection analyzer (Foss Tecator, Högnäs, Sweden).

### 2.5 Statistical analysis

Statistical analyses used SAS software, version 9.3 (SAS Institute Inc., Cary, NC, USA). Simple linear regressions were computed for comparing sheep live weights, plant species richness, plant species evenness, root biomass, soil organic carbon, soil total nitrogen, soil phosphorus in relation to years. ANCOVA was used to test the assumption of homogeneity of regression slopes. The level of significance used was *p* < .05. All figures were constructed using Sigma Plot 12.5 software.

## 3 RESULTS

### 3.1 Sheep weight gain and deaths

**Figure 3:**
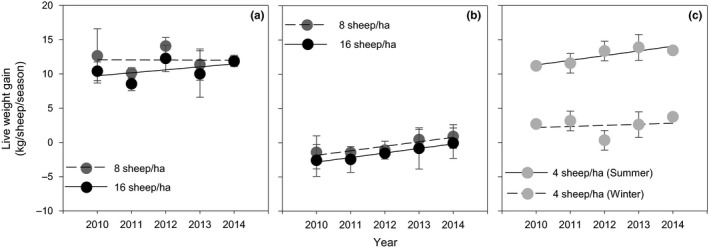
Weight gain of sheep in warm (a), cold (b), and warm + cold (c) season stocking treatments for five consecutive years. Each point is the mean of 48 measurements in the warm and cold season grazing treatments and of 24 measurements in the warm + cold season stocking treatments. Lines are fitted by regression. Bars are *SE*'s

The weight gain of individual sheep in the warm season varied from 8.6 kg/sheep to 14.5 kg/sheep (Figure 3a) and the average weight gain was 10.6 kg at 16 sheep/ha and 12.0 kg at 8 sheep/ha. However, there was no significant difference between the two stocking rates in weight gains. Furthermore, the weight gains at both 8 and 16 sheep/ha did not significantly change over the 5 years of the warm season only stocking. In contrast, sheep lost weight during the first 3 months of the cold season only stocking treatment (Figure 3b). The average weight loss of individual sheep was 1.5 kg at 16 sheep/ha and 0.5 kg at 8 sheep/ha but these were not significant. However, the weight loss during the cold season only stocking decreased significantly over the 5 years; at *p* < .01 for 8 sheep/ha and *p* < .05 for 16 sheep/ha. When sheep were continuously stocked in the warm + cold season treatment at 4 sheep/ha (Figure 3c), they gained weight in a similar manner to that of sheep rotationally stocked in the warm season only stocking treatment at 8 or 16 sheep/ha. The weight gain increased steadily during the 5 years (*p* < .05), suggesting that the quality of the herbage on offer or the diet selected improved during the 5 years. When these same sheep grazed during the early cold season months they gained a small and similar weight during each of the 5 years.

**Figure 4:**
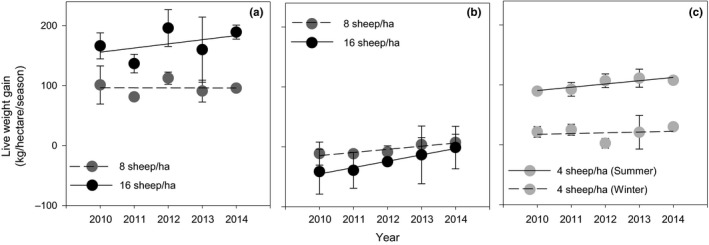
Weight gain of hectare in the warm (a), cold (b), and warm + cold (c) season stocking treatments for five consecutive years. Each point is the mean of 48 measurements in the warm season, cold season stocking treatments, and 24 measurements in the warm + cold season stocking treatments. Lines are fitted by regression. Bars are *SE*s

The weight gain per hectare during the warm season only stocking treatment was significantly higher (*p* < .05) for the 16 than 8 sheep/ha stocking rate and there was no significant change at either stocking rate during the 5 years (Figure 4a). During the cold season, only stocking treatment sheep lost a small weight and the amount lost significantly (*p* < .05) declined over the 5 years (Figure 4b). When sheep were continuously grazed in warm and cold seasons (Figure 4c), the weight gain per hectare in the warm season was similar to that of 8 sheep/ha in the warm season only stocking treatment. In the cold season, the sheep maintained their weight.

During the years 2011 to 2014, no sheep died in the months of June and July. From August to December on average, 1.1% died each month (August to December) from disease and 0.45% from wolf attack.

### 3.2 Plant species richness

**Figure 5:**
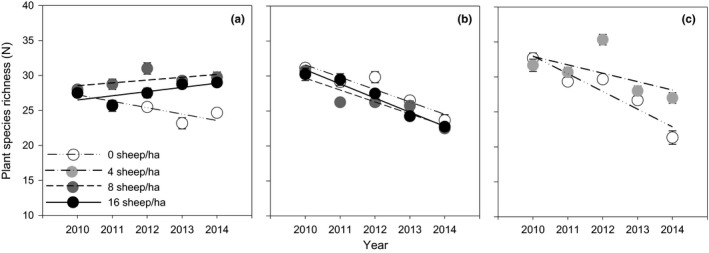
Plant species richness in warm (a), cold (b), and warm + cold (c) season stocking treatments measured in August of each year. Stocking rates were 0, 8, and 16 sheep/ha (a and b) and 0 and 4 sheep/ha (c). Each point is the mean of six measurements. Lines are fitted by regression. Bars are *SE*s

When the grassland was not stocked (control), there was a significant loss of plant species (Figure 5a–c). The rate of loss differed between the three stocking treatments and was significant at *p* < .01, *p* = .01, and *p* < .01, respectively. In contrast, warm season stocking at either 8 or 16 sheep/ha did not significantly alter the plant species richness (Figure 5a). There was a significant (*p* < .01) decline in plant species richness when the grassland was only stocked during the cold season. The rate of decline was about 1.4 species/year for the two cold season only stocking treatments (Figure 5b). When sheep were continuously stocked in both the warm + cold season stocking treatments (Figure 5c), there was no significant change in number of plant species. The species lost were *Stipa aliena*,* Delphinium grandiflorum*,* Swertia franchetiana*,* Gentiana spathulifolia*,* Saussurea pachyneura*,* Leontopodium alpinum*,* Anaphalis lacteal,* and *Plantago asiatica*. These were minor components of the grassland and were of low to medium palatability except for the grass *Stipa aliena* which was of high palatability.

### 3.3 Plant species evenness

**Figure 6:**
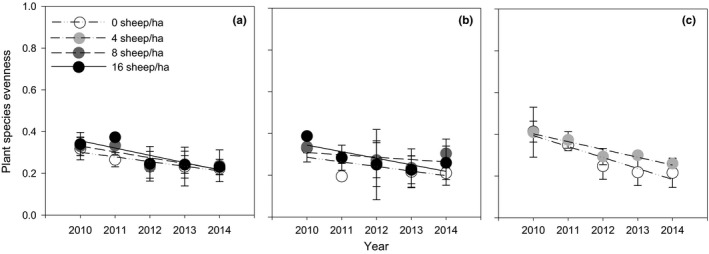
Plant species evenness (Camargo evenness index) in warm (a), cold (b), and warm + cold (c) season stocking treatments measured in August of each year. Stocking rates were 0, 8, and 16 sheep/ha (a and b) and 0 and 4 sheep/ha (c). Each point is the mean of six measurements. Lines are fitted by regression. Bars are *SE*s

The evenness index significantly declined (*p* = .03 to .0001) in all treatments (Figure 6a–c) over the 5 years except for the cold season stocking treatment at 8 sheep/ha (Figure 6b). The slopes were also similar indicating the loss of evenness was at a constant rate.

### 3.4 Root biomass

**Figure 7:**
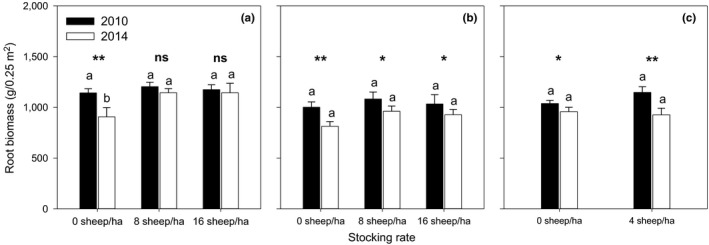
Root biomass weights in the surface 10 cm of soil in warm (a), cold (b), and warm + cold (c) season stocking system measured in 2010 and 2014. Stocking rates were 0, 8, and 16 sheep/ha (a and b) and 0 and 4 sheep/ha (c). Weights are the means (±*SE*) of six measurements. Weights within a stocking season system with different letters are significantly different from each other at the 0.05 level. Significance of differences between each weight in 2010 and 2014 is ns = not significant, * = significant at 0.05 level, ** = significant at 0.01 level

In the nonstock treatment (controls) for each of the three treatments (Figure 7a–c), root biomass declined over the 5 years by 100 to 200 g/0.25 m^2^ from 2010 to 2014. By contrast, there was no significant change in root biomass when stocking was only in the warm season; stocking in the cold season and in both the warm + cold seasons significantly decreased root biomass compared to no stocking.

### 3.5 Soil organic carbon, total nitrogen, and total phosphorus

There were no changes in SOC content and TN content of the surface 10 cm of soil in any treatments (Table 2). There was no change in TP content of the surface 10 cm of soil in warm and warm + cold season stocking treatments (Table 2). In contrast, there was a steady decline of TP in the cold season stocking treatment; this decline was significant (*p* < .05) for the 8 sheep/ha and highly significant (*p* < .01) for 16 sheep/ha stocking rate. No significant change occurred from 2010 to 2016 in SOC, TN or TP contents of soil from the 10 to 40 cm depths and these data are not shown.

**Table 2:** Organic carbon, total nitrogen, and total phosphorus percentages in the surface 10 cm of soil in warm, cold, and warm + cold season grazing treatments measured in 2010 and 2014. Stocking rates were 0, 8, and 16 sheep/ha (warm, cold season grazing) and 0 and 4 sheep/ha (warm + cold season grazing). Each percentage is the mean (±*SE*) of six samples. Significance of differences between each percentage value in 2010 and 2014 is * = significant at 0.05 level, ** = significant at 0.01 level. Where significance is not signified there is no significance

		Warm season stocking		Cold season stocking		Warm + cold season stocking	
Soil properties	Stocking treatments (sheep/ha)	2010	2014	2010	2014	2010	2014
Soil organic carbon	0	97.12 ± 37.69a	99.47 ± 9.45a	95.14 ± 3.68a	94.04 ± 4.64a	91.78 ± 4.54a	95.11 ± 7.83a
	4	—	—	—	—	86.99 ± 3.49a	88.92 ± 4.31a
	8	109.62 ± 3.86a	108.48 ± 8.54a	89.72 ± 4.35a	87.06 ± 2.89a	—	—
	16	116.39 ± 3.41a	104.93 ± 10.92a	108.29 ± 5.65a	100.41 ± 8.22a	—	—
Soil total nitrogen	0	6.20 ± 0.42a	6.06 ± 0.69a	8.31 ± 0.67a	7.49 ± 0.32a	6.45 ± 0.76a	6.44 ± 0.16a
	4	—	—	—	—	7.08 ± 0.84a	6.37 ± 0.76a
	8	6.63 ± 0.57a	6.44 ± 0.94a	8.76 ± 0.72a	7.45 ± 0.15a	—	—
	16	6.72 ± 0.54a	6.42 ± 0.22a	7.21 ± 0.49a	6.49 ± 0.91a	—	—
Soil total phosphorus	0	0.34 ± 0.01a	0.30 ± 0.01a	0.45 ± 0.03a	0.39 ± 0.02a	0.41 ± 0.01a	0.40 ± 0.04a
	4	—	—	—	—	0.37 ± 0.06a	0.39 ± 0.04a
	8	0.38 ± 0.01a	0.37 ± 0.05a	0.46 ± 0.09a*	0.38 ± 0.06a	—	—
	16	0.38 ± 0.06a	0.39 ± 0.06a	0.44 ± 0.03a**	0.33 ± 0.08a	—	—

## 4 DISCUSSION

The stocking systems recommended by the Government of China for the herders of domestic stock on the QTP emerged from practical and general scientific knowledge and political imperatives. This is the first conventional stocking system study available to specifically evaluate the “Retire Livestock and Restore Grassland” policy for the Alpine Meadow grasslands and in doing so, to evaluate the benefits of the policy for Tibetan herders. Resting grassland from stocking during the warm season for later cold season stocking or yearlong resting led to a significant and steady reduction of plant species richness (Figure 5a) and evenness (Figure 6a) and root biomass (Figure 7a) but not soil C, N, and P (Table 2) which remained unchanged. Retiring stock may not therefore fully restore dysfunctional grassland; judicial stocking may be more effective in achieving this goal. The continuous stocking system of four sheep/ha maintained both plant species richness (Figure 5c) and evenness (Figure 6c) and soil C, N, and P (Table 2) content. It was observed that abundant flowering and seed set of grasses and forbs occurred in this stocking system, and there was complete ground cover throughout the warm season. If restoration of grassland functionality is achieved with judicial stocking of allocated land, then the cost/benefit of fencing becomes problematic.

The design and operation of the stocking systems study were limited by availability of valley land that was similar and the extremely adverse operating conditions in the cold season. Although the warm, cold, and warm + cold season treatment(s) were laid out in separate areas which precluded statistical comparison of the full treatment set, it is most likely there was no spatial variation that would significantly influence the outcomes of a statistical analysis of the full set of treatments. Soil (Table 2) and plant species richness and evenness (Figures 5 and 6) of the nonstocked control plots in each set of stocking systems were not different from each other indicating little or no spatial variation. The site available for the stocking treatments was not degraded which precludes a consideration of the effectiveness of the treatments in restoring functionality to these Alpine Meadow grasslands.

### 4.1 Adverse consequences of exclusion of stocking during the warm season

The removal of stock from this grassland during the warm season had adverse consequences for plant conservation. Plant species richness steadily declined (Figure 5a), although the soil attributes measured did not (Table 2). The steady decline in plant species richness when grassland was reserved during the warm season and eaten by sheep during the cold season (Figure 5b) would therefore have occurred for the same reason and not the cold season grazing per se. The plant species lost were minor in the plant community and given that the forb species in general are of low to medium palatability, they were probably outcompeted for light by taller, erect, and more vigorous species. The one grass species lost in all stocking systems (Figure 5a–c) was highly palatable, and so would have been killed by selective grazing by the sheep. These losses of forbs and grass(es) (Figure 5b) could probably be managed by judicious grazing during the warm season.

The stocking systems, including nonstocking during the warm season similarly were associated with a steady decline in plant species evenness. Given the similar decline in all stocking systems (Figure 6a–c), it is highly likely the steady decline has its origin in the shift from yak to sheep stocking. Yaks are much less selective than sheep in their grazing because of their large mouths (Liu et al., 2015). The sheep may have selectively grazed some or all of the dominant species that yak would have not have and selectively grazed out some minor species thereby lowering the evenness of plant species. As a similar decrease in plant species evenness occurred in the nonstocked treatment (control), it is probable that species differences in plant architectures in both stocked and nonstocked grassland may have been involved. Removal of stock would allow naturally tall and “bulky” species to dominate thereby decreasing plant species evenness.

The steady loss of plant species richness by resting grasslands during the warm season does not support the government policy of “Retire Livestock and Restore Grassland.” Survey of stocked and nonstocked grassland in the Maqu County also indicated lower plant species richness in nonstocked compared with stocked grassland (Wu, Du, Liu, & Thirgood, 2009). Confirmation of plant species loss by removing stock during the warm season has implications for both restoration and stock production. To restore degraded grassland, stocking appears to be required but the rate of stocking which fosters restoration needs to be established by a long‐term stocking rate study involving a range of rates from low to very high rates on nearby dysfunctional and functional landscapes. To maintain stock production, the stocking system should include stocking during the warm seasons. Not to do so would predispose loss of animal productivity as animal production declines with lowering of plant species richness in the diet of sheep (Wang, Wang, et al., 2010; Wang, Zhao, et al., 2010).

### 4.2 Sustainability of continuous stocking

The cessation of stocking of sheep or yak on the QTP by exclusion fencing has been linked to increases in soil carbon, phosphorus, and nitrogen (Dong et al., 2012; Wu, Liu, Zhang, Chen, & Hu, 2010) but this was not confirmed in this study. Five years of warm or cold season stocking and warm + cold season stocking did not significantly change the carbon, nitrogen, phosphorus, or root biomass levels in the surface soil. This finding indicates that sustainable sheep production at the stocking rates applied in our study (up to 16 sheep/ha) in both the warm and cold season pastures can be achieved without adverse change to the soil environment. This conclusion differs from that of others and cannot readily be reconciled. Although there was no apparent change in soil properties from exclusion of stock during the warm season to produce “reserved” pasture for cold season stocking in our study, the steady loss of plant species remains a concern.

The common practice of continuous stocking in both warm and cold seasons is considered to be unsustainable by some researchers (e.g., Miao et al., 2015). However, in our continuous stocking treatment, there were no indications of adverse change in sheep production, plant species, or soil properties that would indicate an unsustainable practice. To the contrary, the sheep production per individual (and hectare) in the warm season steadily and significantly increased during the 5 years suggesting improved functionality of the grassland (Figure 4a). At 4 sheep/ha, there was considerable biomass of grass remaining in December which would have been consumed later in the cold season had stocking continued through this period. Although our data strongly supports 4 sheep/ha as being sustainable, there was only one stocking rate applied so we are unable to determine the critical threshold in stocking rate beyond which the grassland resource degrades if continuous grazing is practiced. We could not find in the literature any economic analyses of continuous stocking management.

### 4.3 Sustainability of warm season only stocking and cold season housing

An alternative to continuous stocking at a modest stocking rate is to only stock the grassland during the 4 months of the warm season and then house and hand feed stock for the next 8 months. Our data for warm season only stocking indicate that up to 16 sheep/ha can be sustainably stocked. This is almost certainly at the peak of animal productivity/ha and profitability for the grassland at the study site (Sun et al., 2015). In a comparison of the economics of grazing versus hand feeding of housed animals (Zhao et al., 2010) in the northern edge of the QTP, hand‐fed and housed sheep in the 6 months of December to May made a net profit per animal during this period of 5.4, 45.7, and 59.7 Renminbi (RMB) for older ewes, young ewes, and lambs, respectively. In contrast older ewes and young ewes when stocked on grassland, lost values of −28.3 and −13.1 RMB per sheep, respectively, and lambs gained value of 29.3 RMB. Housed sheep were hand‐fed locally produced maize cobs, alfalfa and oat hay, beer‐making residue and onions. We conclude that warm season stocking, coupled with hand feeding of cold season housed sheep, is a more profitable sheep management model than continuous stocking and deserves further economic analysis to confirm this view.

The management of grazing and supplementary feeding during the cold season is a critical part of the sheep production systems on the QTP. Sheep cannot survive the intense cold and chilling winds at night on the grasslands, and wolf predation is a constant threat unless they are in a yard and watched. Nighttime sheltering of sheep in the cold season is essential for sustainable production. In this study, the sheep were returned to open traditional sheep yards at night, but not fed, after daytime grazing. These sheep did not gain weight and sometimes lost weight during the cold season (Figure 3b), and stocking rate did not affect their weight change. Nighttime housing in enclosed sheds would raise weight gains and reduce deaths. Supplementary feeding during nighttime would further raise weight gains but the economic benefits need to be assessed against the cost of the feed (Xu et al., 2017; Yang et al., 2011). In a study by Yang et al. (2011), cold season feeding options were modeled; stocking sheep on grassland without supplementary feed at night was found to be the most profitable option for the cold season.

### 4.4 Sustainability of stocking for “optimal” profitability

The stocking rate in the warm season will determine both the productivity of the sheep and the profitability, as supplementary feeding and other management costs will be fixed and linearly related to the number of sheep in the pastoral business. If it is assumed that individual sheep production declines linearly with increase in stocking rate, that housing for sheep is available and that feeding and management costs are fixed, then the stocking rate during the warm season in this environment should be about 16 sheep/ha to maintain the grassland resource and to maximize the profitability from the stocked land (see Sun et al., 2015). Raising the sheep stocking rate in the warm season beyond 16 sheep/ha would increase the probability of the pastoral business crossing a critical threshold beyond which economic recovery and restoration of the grassland becomes problematic (Ash & McIvor, 2005).

### 4.5 Implications for Tibetan herders

The stocking experiment and its results pertain to the settlement model for herders. This model has the advantage of creating a large enough community to attract education facilities, small businesses, and other services. There are, however, other successful and possible models (Shang et al., 2014) such as the pure nomadic model and semi‐settlement models in between. Resolving the conflict between forage and livestock production in the context of sustainability, socio‐economic systems, off‐farm employments, markets, etc. is extremely complex. Shang et al. (2014) identified and reviewed 18 strategies currently practiced on the QTP to achieve sustainable livelihoods for its pastoral people. No single model will suffice and national support by policy and investment, local and regional commitment to capacity building, and the involvement of herders need to be continued and strengthened.

## CONFLICT OF INTEREST

None declared.

## AUTHOR CONTRIBUTION

Fujiang Hou designed and supervised the project. Yingxin Wang, Zhaofeng Wang, and Shenghua Chang conducted the field work and collected the data. Yingxin Wang, Ken Hodgkinson, and Fujiang Hou wrote the manuscript with critical input from all the authors.

## References

[ece33960-bib-0001] Alkemade, R. , Reid, R. S. , van den Berg, M. , de Leeuw, J. , & Jeuken, M. (2013). Assessing the impacts of livestock production on biodiversity in rangeland ecosystems. Proceedings of the National Academy of Sciences of the United States of America, 110, 20900–20905. https://doi.org/10.1073/pnas.1011013108 22308313 10.1073/pnas.1011013108 PMC3876242

[ece33960-bib-0002] Allen, V. G. , Batello, C. , Berretta, E. J. , Hodgson, J. , Kothmann, M. , Li, X. L. , & Sanderson, M. (2011). An international terminology for grazing lands and grazing animals. Grass and Forage Science, 66, 2–28. https://doi.org/10.1111/j.1365-2494.2010.00780.x

[ece33960-bib-0003] Andersson, E. , Brogaard, S. , & Olsson, L. (2011). The political ecology of land degradation. Annual Review of Environment and Resources, 36, 295–319. https://doi.org/10.1146/annurev-environ-033110-092827

[ece33960-bib-0004] Ash, A. J. , & McIvor, J. G. (2005). Constraints to pastoral systems in marginal environments In Pastoral systems in marginal environments, Proceedings of a satellite workshop of the XXth International Grassland Congress, pp. 17–28.

[ece33960-bib-0005] Bedunah, D. J. , & Angerer, J. P. (2012). Rangeland degradation, poverty, and conflict: How can rangeland scientists contribute to effective responses and solutions? Rangeland Ecology and Management, 65, 606–612. https://doi.org/10.2111/REM-D-11-00155.1

[ece33960-bib-0006] Briske, D. D. , Derner, J. D. , Brown, J. R. , Fuhlendorf, S. D. , Teague, W. R. , Havstad, K. M. , & Willms, W. D. (2008). Rotational grazing on rangelands: Reconciliation of perception and experimental evidence. Rangeland Ecology & Management, 61, 3–17. https://doi.org/10.2111/06-159R.1

[ece33960-bib-0007] Brondizio, E. S. , & Le Tourneau, F. M. (2016). Environmental governance for all. Science, 352, 1272–1273. https://doi.org/10.1126/science.aaf5122 27284179 10.1126/science.aaf5122

[ece33960-bib-0008] Camargo, J. A. (1993). Must dominance increase with the number of subordinate species in competitive interactions? Journal of Theoretical Biology, 161, 537–542. https://doi.org/10.1006/jtbi.1993.1072

[ece33960-bib-0009] Cao, J. J. , Xiong, Y. C. , Sun, J. , Xiong, W. F. , & Du, G. Z. (2011). Differential benefits of multi‐and single‐household grassland management patterns in the Qinghai‐Tibetan plateau of China. Human Ecology, 39, 217–227. https://doi.org/10.1007/s10745-011-9384-0

[ece33960-bib-0010] Chen, H. , Zhu, Q. , Peng, C. H. , Wu, N. , Wang, Y. F. , Fang, X. Q. , … Wu, J. H. (2013). The impacts of climate change and human activities on biogeochemical cycles on the Qinghai‐Tibetan Plateau. Global Change Biology, 19, 2940–2955. https://doi.org/10.1111/gcb.12277 23744573 10.1111/gcb.12277

[ece33960-bib-0011] Chinese Soil Taxonomy Research Group (1995). Chinese soil taxonomy (pp. 58–147). Beijing, China: Science Press.

[ece33960-bib-0012] Dong, Q. M. , Zhao, X. Q. , Wu, G. L. , & Chang, X. F. (2015). Optimization yak grazing stocking rate in an alpine grassland of Qinghai‐Tibetan Plateau, China. Environmental Earth Sciences, 73, 2497–2503. https://doi.org/10.1007/s12665-014-3597-7

[ece33960-bib-0013] Dong, Q. M. , Zhao, X. Q. , Wu, G. L. , Shi, J. J. , Wang, Y. L. , & Sheng, L. (2012). Response of soil properties to yak grazing intensity in a *Kobresia parva*‐meadow on the Qinghai‐Tibetan Plateau, China. Journal of Soil Science and Plant Nutrition, 12, 535–546.

[ece33960-bib-0014] Du, W. C. , Yan, T. , Chang, S. H. , Wang, Z. F. , & Hou, F. J. (2017). Seasonal hogget grazing as a potential alternative grazing system for the Qinghai‐Tibetan plateau: Weight gain and animal behaviour under continuous or rotational grazing at high or low stocking rates. The Rangeland Journal, 39, 329–339.

[ece33960-bib-0015] Hao, X. (2008). A green fervor sweeps the Qinghai‐Tibetan plateau. Science, 321, 633–635. 18669838 10.1126/science.321.5889.633

[ece33960-bib-0016] Harris, R. B. (2010). Rangeland degradation on the Qinghai‐Tibetan plateau: A review of the evidence of its magnitude and causes. Journal of Arid Environments, 74, 1–12. https://doi.org/10.1016/j.jaridenv.2009.06.014

[ece33960-bib-0017] Harris, R. B. , Wang, W. Y. , Smith, A. T. , & Bedunah, D. J. (2015). Herbivory and competition of Tibetan steppe vegetation in Cold season pasture: Effects of livestock exclosure and plateau pika reduction. PLoS ONE, 10, e0132897 https://doi.org/10.1371/journal.pone.0132897 26208005 10.1371/journal.pone.0132897 PMC4514881

[ece33960-bib-0018] Jones, R. J. , & Sandland, R. L. (1974). The relation between animal gain and stocking rate: Derivation of the relation from the results of grazing trials. The Journal of Agricultural Science, 83, 335–342. https://doi.org/10.1017/S0021859600052035

[ece33960-bib-0019] Kang, L. , Han, X. G. , Zhang, Z. B. , & Sun, O. J. (2007). Grassland ecosystems in China: Review of current knowledge and research advancement. Philosophical Transactions of the Royal Society of London B: Biological Sciences, 362, 997–1008. https://doi.org/10.1098/rstb.2007.2029 17317645 10.1098/rstb.2007.2029 PMC2435566

[ece33960-bib-0020] Kemp, D. R. , Han, G. D. , Hou, X. Y. , Michalk, D. L. , Hou, F. J. , Wu, J. P. , & Zhang, Y. J. (2013). Innovative grassland management systems for environmental and livelihood benefits. Proceedings of the National Academy of Sciences of the United States of America, 110, 8369–8374. https://doi.org/10.1073/pnas.1208063110 23671092 10.1073/pnas.1208063110 PMC3666733

[ece33960-bib-0021] Lehnert, L. W. , Wesche, K. , Trachte, K. , Reudenbach, C. , & Bendix, J. (2016). Climate variability rather than overstocking causes recent large scale cover changes of Tibetan pastures. Scientific Reports, 6, 24367 https://doi.org/10.1038/srep24367 27073126 10.1038/srep24367 PMC4829870

[ece33960-bib-0022] Li, X. L. , Gao, J. , Brierley, G. , Qiao, Y. M. , Zhang, J. , & Yang, Y. W. (2013). Rangeland degradation on the Qinghai‐Tibet plateau: Implications for rehabilitation. Land Degradation & Development, 24, 72–80. https://doi.org/10.1002/ldr.1108

[ece33960-bib-0023] Liu, J. , Feng, C. , Wang, D. L. , Wang, L. , Wilsey, B. J. , & Zhong, Z. W. (2015). Impacts of grazing by different large herbivores in grassland depend on plant species diversity. Journal of Applied Ecology, 52, 1053–1062. https://doi.org/10.1111/1365-2664.12456

[ece33960-bib-0024] Liu, J. G. , Li, S. X. , Ouyang, Z. Y. , Tam, C. , & Chen, X. D. (2008). Ecological and socioeconomic effects of China's policies for ecosystem services. Proceedings of the National Academy of Sciences of the United States of America, 105, 9477–9482. https://doi.org/10.1073/pnas.0706436105 18621700 10.1073/pnas.0706436105 PMC2474515

[ece33960-bib-0025] Lu, X. Y. , Kelsey, K. C. , Yan, Y. , Sun, J. , Wang, X. D. , Cheng, G. W. , & Neff, J. C. (2017). Effects of grazing on ecosystem structure and function of alpine grasslands in Qinghai‐Tibetan Plateau: A synthesis. Ecosphere, 8, 1–15.29552374

[ece33960-bib-0026] Ludwig J. A., Tongway D. J., Freudenberger D. O., Noble J. C., & Hodgkinson K. C. (Eds.) (1997). Landscape ecology, function and management: Principles from Australia's rangelands. Clayton, Vic.: CSIRO Publishing.

[ece33960-bib-0027] Miao, F. H. , Guo, Z. G. , Xue, R. , Wang, X. Z. , & Shen, Y. Y. (2015). Effects of grazing and precipitation on herbage biomass, herbage nutritive value, and yak performance in an Alpine Meadow on the Qinghai‐Tibetan Plateau. PLoS ONE, 10, e0127275 https://doi.org/10.1371/journal.pone.0127275 26039322 10.1371/journal.pone.0127275 PMC4454548

[ece33960-bib-0028] Miller, D. J. (1999). Nomads of the Tibetan Plateau rangelands in western China. Part Two. Pastoral production practices. Rangelands Archives, 21, 16–19.

[ece33960-bib-0029] Nelson, D. W. , & Sommers, L. (1982). Total carbon, organic carbon, and organic matter. Part 2. Chemical and microbiological properties (methodsofsoilan2). In Page A. L. (Ed.), Methods of soil analysis (pp. 539–579). Madison, WI: American Society of Agronomy.

[ece33960-bib-0030] Noojipady, P. , Prince, S. D. , & Rishmawi, K. (2015). Reductions in productivity due to land degradation in the drylands of the southwestern United States. Ecosystem Health and Sustainability, 1, 1–15. https://doi.org/10.1890/EHS15-0020.1

[ece33960-bib-0031] Pang, X. P. , & Guo, Z. G. (2017). Plateau pika disturbances alter plant productivity and soil nutrients in alpine meadows of the Qinghai‐Tibetan Plateau, China. The Rangeland Journal, 39, 133–144. https://doi.org/10.1071/RJ16093

[ece33960-bib-0032] Pech, R. P. , Arthur, A. D. , Zhang, Y. M. , & Lin, H. (2007). Population dynamics and responses to management of plateau pikas Ochotona curzoniae. Journal of Applied Ecology, 44, 615–624. https://doi.org/10.1111/j.1365-2664.2007.01287.x

[ece33960-bib-0033] Qiu, J. (2007). Environment: Riding on the roof of the world. Nature, 449, 398–402. https://doi.org/10.1038/449398a 17898743 10.1038/449398a

[ece33960-bib-0034] Qiu, J. (2014). Double threat for Tibet. Nature, 512, 240–241. https://doi.org/10.1038/512240a 25143093 10.1038/512240a

[ece33960-bib-0035] Qiu, J. (2016). Trouble in Tibet. Nature, 529, 142–145. https://doi.org/10.1038/529142a 26762440 10.1038/529142a

[ece33960-bib-0036] Ren, J. Z. , Hu, Z. Z. , Zhao, J. , Zhang, D. G. , Hou, F. J. , Lin, H. L. , & Mu, X. D. (2008). A grassland classification system and its application in China. The Rangeland Journal, 30, 199–209. https://doi.org/10.1071/RJ08002

[ece33960-bib-0037] Shang, Z. H. , Gibb, M. J. , Leiber, F. , Ismail, M. , Ding, L. M. , Guo, X. S. , & Long, R. J. (2014). The sustainable development of grassland‐livestock systems on the Tibetan plateau: Problems, strategies and prospects. The Rangeland Journal, 36, 267–296. https://doi.org/10.1071/RJ14008

[ece33960-bib-0039] Smith, A. T. , & Foggin, J. M. (1999). The plateau pika (*Ochotona curzoniae*) is a keystone species for biodiversity on the Tibetan plateau. Animal Conservation, 2, 235–240. https://doi.org/10.1111/j.1469-1795.1999.tb00069.x

[ece33960-bib-0040] Smith, D. M. S. , McKeon, G. M. , Watson, I. W. , Henry, B. K. , Stone, G. S. , Hall, W. B. , & Howden, S. M. (2007). Learning from episodes of degradation and recovery in variable Australian rangelands. Proceedings of the National Academy of Sciences of the United States of America, 104, 20690–20695. https://doi.org/10.1073/pnas.0704837104 18093932 10.1073/pnas.0704837104 PMC2410064

[ece33960-bib-0041] Smith, B. , & Wilson, J. B. (1996). A consumer's guide to evenness indices. Oikos, 76, 70–82. https://doi.org/10.2307/3545749

[ece33960-bib-0042] Steinfeld H., Mooney H. A., Schneider F., & Neville L. E. (Eds.) (2013). Livestock in a changing landscape, Volume 1: Drivers, consequences, and responses. Washington, DC: Island Press.

[ece33960-bib-0043] Sun, Y. , Angerer, J. P. , & Hou, F. J. (2015). Effects of grazing systems on herbage mass and liveweight gain of Tibetan sheep in Eastern Qinghai‐Tibetan Plateau, China. The Rangeland Journal, 37, 181–190.

[ece33960-bib-0044] Wang, X. X. , Dong, S. K. , Sherman, R. , Liu, Q. R. , Liu, S. L. , Li, Y. Y. , & Wu, Y. (2015). A comparison of biodiversity–ecosystem function relationships in alpine grasslands across a degradation gradient on the Qinghai‐Tibetan Plateau. The Rangeland Journal, 37, 45–55. https://doi.org/10.1071/RJ14081

[ece33960-bib-0045] Wang, P. , Lassoie, J. P. , Morreale, S. J. , & Dong, S. K. (2015). A critical review of socioeconomic and natural factors in ecological degradation on the Qinghai‐Tibetan Plateau, China. The Rangeland Journal, 37, 1–9. https://doi.org/10.1071/RJ14094

[ece33960-bib-0046] Wang, L. , Wang, D. L. , He, Z. B. , Liu, G. F. , & Hodgkinson, K. C. (2010). Mechanisms linking plant species richness to foraging of a large herbivore. Journal of Applied Ecology, 47, 868–875. https://doi.org/10.1111/j.1365-2664.2010.01837.x

[ece33960-bib-0048] Wang, M. P. , Zhao, C. Z. , Long, R. J. , & Yang, Y. H. (2010). Rangeland governance in China: Overview, impacts on Sunan County in Gansu Province and future options. The Rangeland Journal, 32, 155–163. https://doi.org/10.1071/RJ09085

[ece33960-bib-0049] Welchons, C. A. , Bondurant, R. G. , Hilscher, F. H. , MacDonald, J. C. , Klopfenstein, T. J. , & Watson, A. K. (2017). Effect of continuous or rotational grazing on growing steer performance and land production. Journal of Animal Science, 95, 170 https://doi.org/10.2527/asasmw.2017.349

[ece33960-bib-0050] Wen, L. , Dong, S. K. , Li, Y. Y. , Li, X. Y. , Shi, J. J. , Wang, Y. L. , … Ma, Y. S. (2013). Effect of degradation intensity on grassland ecosystem services in the alpine region of Qinghai‐Tibetan Plateau, China. PLoS ONE, 8, e58432 https://doi.org/10.1371/journal.pone.0058432 23469278 10.1371/journal.pone.0058432 PMC3587591

[ece33960-bib-0051] Westoby, M. , Walker, B. H. , & Noy‐Meir, I. (1989). Opportunistic management for rangelands not at equilibrium. Journal of Range Management, 42, 266–274. https://doi.org/10.2307/3899492

[ece33960-bib-0052] Wilson, A. D. , & MacLeod, N. D. (1991). Overgrazing: Present or absent? Journal of Range Management, 44, 475–482. https://doi.org/10.2307/4002748

[ece33960-bib-0053] Wu, G. L. , Du, G. Z. , Liu, Z. H. , & Thirgood, S. (2009). Effect of fencing and grazing on a Kobresia‐dominated meadow in the Qinghai‐Tibetan Plateau. Plant and Soil, 319, 115–126. https://doi.org/10.1007/s11104-008-9854-3

[ece33960-bib-0054] Wu, G. L. , Liu, Z. H. , Zhang, L. , Chen, J. N. , & Hu, T. N. (2010). Long‐term fencing improved soil properties and soil organic carbon storage in an alpine meadow swamp of western China. Plant and Soil, 332, 331–337. https://doi.org/10.1007/s11104-010-0299-0

[ece33960-bib-0055] Xu, T. W. , Xu, S. X. , Hu, L. Y. , Zhao, N. , Liu, Z. , Ma, L. , … Zhao, X. (2017). Effect of dietary types on feed intakes, growth performance and economic benefit in Tibetan sheep and Yaks on the Qinghai‐Tibet Plateau during cold season. PLoS ONE, 12, e0169187 https://doi.org/10.1371/journal.pone.0169187 28056054 10.1371/journal.pone.0169187 PMC5215856

[ece33960-bib-0056] Yang, L. , Wu, J. P. , Jones, R. , Kemp, D. , Ma, Z. F. , & Takahashi, T. (2011). Changing livestock and grassland management to improve the sustainability and profitability of alpine grasslands in Sunan county, Gansu province. Development of sustainable livestock systems on grasslands in North‐western China’. ACIAR Proceedings, 134, 69–79.

[ece33960-bib-0057] You, Q. G. , Xue, X. , Peng, F. , Xu, M. H. , Duan, H. C. , & Dong, S. Y. (2014). Comparison of ecosystem characteristics between degraded and intact alpine meadow in the Qinghai‐Tibetan Plateau, China. Ecological Engineering, 71, 133–143. https://doi.org/10.1016/j.ecoleng.2014.07.022

[ece33960-bib-0058] Zhao, H. D. , Liu, S. L. , Dong, S. K. , Su, X. K. , Wang, X. X. , Wu, X. Y. , … Zhang, X. (2015). Analysis of vegetation change associated with human disturbance using MODIS data on the rangelands of the Qinghai‐Tibet Plateau. The Rangeland Journal, 37, 77–87. https://doi.org/10.1071/RJ14061

[ece33960-bib-0059] Zhao, H. J. , Yang, L. , Yang, S. W. , Hua, L. M. , Feng, M. T. , Ma, Z. F. , … Wu, J. P. (2010). Comparison of feeding effect on Gansu alpine fine wool sheep between grazing and greenhouse feeding during withered season. Partacultural Science, 27, 117–121. (In Chinese.)

